# Human Skin Microbiota: High Diversity of DNA Viruses Identified on the Human Skin by High Throughput Sequencing

**DOI:** 10.1371/journal.pone.0038499

**Published:** 2012-06-19

**Authors:** Vincent Foulongne, Virginie Sauvage, Charles Hebert, Olivier Dereure, Justine Cheval, Meriadeg Ar Gouilh, Kevin Pariente, Michel Segondy, Ana Burguière, Jean-Claude Manuguerra, Valérie Caro, Marc Eloit

**Affiliations:** 1 Institut National de la Santé et de la Recherche Médicale U1058- University of Montpellier I- Montpellier University Hospital, Department of Biology and Pathology-Laboratory of Virology and Department of Dermatology, Montpellier, France; 2 Institut Pasteur, Laboratory for Urgent Responses to Biological Threats, Paris, France; 3 Pathoquest, Paris, France; 4 Institut Pasteur, Genotyping of Pathogens and Public Health Platform, Paris, France; 5 Ecole Nationale Vétérinaire d’Alfort, UMR 1161 Virologie ENVA, INRA, ANSES, Maisons Alfort, France; 6 Institut Pasteur, Department of Virology, Paris, France; Ohio State University Medical Center, United States of America

## Abstract

The human skin is a complex ecosystem that hosts a heterogeneous flora. Until recently, the diversity of the cutaneous microbiota was mainly investigated for bacteria through culture based assays subsequently confirmed by molecular techniques. There are now many evidences that viruses represent a significant part of the cutaneous flora as demonstrated by the asymptomatic carriage of beta and gamma-human papillomaviruses on the healthy skin. Furthermore, it has been recently suggested that some representatives of the *Polyomavirus* genus might share a similar feature. In the present study, the cutaneous virome of the surface of the normal-appearing skin from five healthy individuals and one patient with Merkel cell carcinoma was investigated through a high throughput metagenomic sequencing approach in an attempt to provide a thorough description of the cutaneous flora, with a particular focus on its viral component. The results emphasize the high diversity of the viral cutaneous flora with multiple polyomaviruses, papillomaviruses and circoviruses being detected on normal-appearing skin. Moreover, this approach resulted in the identification of new *Papillomavirus* and *Circovirus* genomes and confirmed a very low level of genetic diversity within human polyomavirus species. Although viruses are generally considered as pathogen agents, our findings support the existence of a complex viral flora present at the surface of healthy-appearing human skin in various individuals. The dynamics and anatomical variations of this skin virome and its variations according to pathological conditions remain to be further studied. The potential involvement of these viruses, alone or in combination, in skin proliferative disorders and oncogenesis is another crucial issue to be elucidated.

## Introduction

Skin represents the main barrier between external, potentially aggressive agents and inner, more vulnerable structures. Apart from the physical protection based on epidermis cohesion and more particularly to its cornified layer, the presence of a complex ecosystem plays a well-documented role in preventing adherence and invasion by either bacterial or fungal virulent agents through biological competition and occupation of favorable ecological positions. Indeed, skin harbors various physiological populations of micro-organisms including commensal or symbiotic bacteria, fungi, parasites and viruses, overall known as skin microbiota [1]. Numerous non pathogenic skin bacteria were historically described through culture isolation methods and the wide diversity of this cutaneous flora was recently confirmed by molecular approaches [2]. More specifically, the complexity of the skin bacterial microbiota was mostly analyzed through deep sequence analysis of the universal 16S small subunit ribosomal gene in procaryotes. Conversely, the skin viral microbiota was more rarely investigated partly because most skin-associated viruses are not cultivable and do not display such consensus sequences that can be targeted by high input molecular methods. There is now a growing corpus of evidence that healthy-looking human skin also harbors resident or transient viruses. The validity of this concept was extensively demonstrated for cutaneous beta and gamma human papillomaviruses (β and γ-HPVs) that are commonly present on the superficial layers of the skin in most individuals [3]–[5]. Indeed, in this setting, several studies have reported the genetic diversity of cutaneous human papillomaviruses using consensus PCR targeting L1 gene [6] or more recently through sequencing rolling circle products [7], [8]. Furthermore, functional metagenomic methods were recently applied to cutaneous samples and led to the description of new human virus species belonging to the *Polyomaviridae* family. One of the most striking illustrations of these findings is the recent identification of the Merkel cell polyomavirus (MCPyV), primarily isolated from an aggressive neuro-endocrine skin tumor [9], [10], the Merkel cell carcinoma (MCC), but subsequently detected on the skin surface of most healthy individuals, as well as on normal or pathological skin of patients with benign or non–MCC malignant skin conditions, thus raising the question of its genuine involvement in skin oncogenesis [11]–[13]. More recently, two additional representatives of the human polyomavirus (HPyV) genus, named HPyV6 and HPyV7, were identified on the skin surface of healthy individuals [12].

In an attempt to provide a thorough description of the physiological skin viral microbiota, the cutaneous virome of the surface of the normal-appearing skin in five healthy individuals and in one patient with MCC was investigated using a high throughput metagenomic sequencing (HTS) approach. Overall, this study confirms the high diversity of the viral component of the skin microbiota and suggests the existence of a resident cutaneous viral flora, the pattern of which is possibly different according to skin conditions. The physiological importance of this viral microbiota remains to be established and may encompass varied functions such as a direct anti-bacterial or anti-fungal action or an indirect protection against other, more aggressive virus through competition mechanisms by analogy with the skin bacterial microbiota.

## Results

### Sequence Depth, Assembly and Taxonomic Assignment

The Illumina Sequencing was conducted with a mean depth per sample of 8.6×10^6^ (range 7.6–10.3×10^6^) paired-ends reads of 100 nucleotides (nt) size. After human genome sequence subtraction through SOAP and BlastN, an average of 4.4×10^6^ (range 1.5–7.6×10^6^) reads per sample was analyzed for contig assembly and assignment. An average of 96.1% (range 91.1–98.9%) of the 100 nt reads could be assembled. The majority of identified contigs and of the remaining 100 nt singletons were assigned through both BlastN and BlastX programs. The upper cutoff value for taxonomic assignment was set to10^−3^ (E<10^−3^). All related data are detailed in Table 1.

**Table 1:** Workflow of the sequences analysis, from raw data to assignment.

Samples	100066			100067			100069		100070		100072			100073		
Total reads, raw data	8,052,770			10,354,496			9,107,144		8,196,240		7,588,712			10,281,130		
Total reads, Soap and BlastN filtered	2,849,108 (100%)			4,556,951 (100%)			7,690,427 (100%)		1,572,456 (100%)		4,422,817 (100%)			5,255,640 (100%)		
**Sequence assembly:** CLC Genomics Workbench																
Contig number (reads number)	9,242 (2,786,250)		105,232 (4,228,142)			25,563 (7,521,632)			87,576 (1,432,804)			11,765 (4,348,806)		13,948 (5,196,480)		
% total reads	97.8%		92.8%			97.8%			91.1%			98.3%		98.9%		
Singletons (Unassembled reads)	62,858		328,809			168,795			139,652			74,011		59,160		
% total reads	2.2%		7.2%			2.2%			8. 9%			1.7%		1.1%		
**Taxonomic assignment:** Blast search (BlastN and BlastX) within NCBI database (E value <10^−3^)																
% Assigned reads, contigs + singletons(reads number)		99.7% (2,841,768)			90.6% (4,131,010)			99.4% (7,646,231)		94.1% (1,480,258)			98.6% (4,359,580)		99.5% (5,229,528)	
Contig number (reads number)		8,521 (2,783,976)			70,765 (3,950,373)			21,595 (7,488,486)		67,204 (1,374,873)			10,898 (4,317,823)		13,107 (5,176,684)	
Contig average lenght (nt)		216			197			191		157			309		219	
% Assigned singletons (reads number)		2.0% (57,792)			3.9% (180,637)			2.0% (157,745)		6.7% (105,385)			0.9% (41,757)		1.0% (52,844)	
**Unassigned reads:** Blast search (BlastN and BlastX) within NCBI database (E value <10^−3^)																
% Unassigned reads, contigs + singletons (reads number)		0.3% (7,340)			9.4% (425,941)			0.6% (44,196)		5.9% (92,198)			1.4% (63,237)		0.5% (26,112)	
Contig number (reads number)		721 (2,274)			34,567 (277,769)			3,968 (33,146)		20,372 (57,931)			867 (30,983)		841 (19,796)	
Contig average lenght (nt)		138			143			125		126			162		144	
Contig maximum lenght (nt)		975			1,396			1,480		1,665			2,079		3,139	
Singletons		5,066			148,172			11,050		34,267			32,254		6,316	

Assignment results were categorized according to bacterial sequences, with further distinction between Archae and Eubacteria, or plasmids and viruses sequences. Viral sequences were further classified between procaryotic viruses (phages) and eucaryotic DNA viruses. The remaining sequences were either related to eucaryotic organisms (Fungi, Viridiplantae, Protozoa and Metazoa) or were falling in the miscellaneous "others" group that included unclassified sequences, endogenous retroviral sequences, RNA virus sequences, sequences identified by previous freshwater, marine or human gut metagenomes studies and vectors derived sequences. As displayed in Table 2, most of the sequences were identified as belonging to the Eubacteria and eucaryotic DNA viruses groups, although with large inter individual differences in the proportion of these two groups.

**Table 2:** Relative proportion of the cutaneous microbiome in each sample.

Samples	100066	100067	100069	100070	100072	100073
Total reads, Soap andBlastN filtered	**2,849,108**	**4,556,951**	**7,690,427**	**1,572,456**	**4,422,817**	**5,255,640**
**Assignment** % total reads (reads number)						
Archea	**<0.1%** (122)	**0.1%**(3,610)	**<0.1%** (220)	**<0.1%** (526)	**0.4%**(17,660)	**<0.1%** (251)
Eubacteria	**23.1%** (658,075)	**60,7%** (2,766,794)	**8.7%** (672,919)	**57.2%** (900,033)	**65.0%** (2,876,654)	**67.3%** (3,537,704)
Plasmids	**0.3%** (9,750)	**0.5%** (22,233)	**0.3%** (22,198)	**1.5%** (23,226)	**2.9%** (129,944)	**2.4%** (128,941)
Phages	**<0.1%** (235)	**0.3%**(12,167)	**<0.1%** (187)	**5.4%** (84,750)	**0.3%** (14,746)	**0.8%** (41,532)
Eukaryotes*	**2.7%** (78,235)	**13.6%** (619,289)	**3.1%** (235,257)	**22.4%** (352,549)	**6.5%** (28,7,559)	**6.1%** (320,590)
Other**	**0.5%** (15,008)	**1.6%** (74,572)	**0.2%** (16,734)	**0.7%** (10,746)	**0.7%** (31,084)	**0.9%** (47,682)
Eukaryotic DNA virus	**73.0%** (2,080,343)	**13.9%** (632,345)	**87.1%** (6,698,716)	**6.9%** (108,428)	**22.6%** (1,001,937)	**21.9%** (1,152,828)
Unassigned reads	**0.3%** (7,340)	**9.3%** (425,941)	**0.6%** (44,196)	**5.9%** (92,198)	**1.4%** (63,233)	**0,5%** (26,112)

-*Eukaryotes denotes *Fungi*, *Viridiplantae*, *Protozoa* and *Metazoa* sequences.

- **Other denotes metagenome, vectors, RNA virus, endogenous retrovirus and unclassified sequences.

### Bacterial Sequence Assignment

An average of 47% (median, 58.9%; first quartile, 23%; third quartile, 65%) of the sequences could be assigned to bacterial sequences. Sequences were classified according to the best hit sequence in their corresponding phylum. Sequences from the Firmicutes (mainly *Staphylococcus* and *Streptococcus* genera), the Actinobacteria (mainly *Corynebacterium* and *Propionibacterium* genera), the Proteobacteria, and the Bacteroidetes phyla were the more widely represented among the 6 samples. Bacterial sequences belonging to the Cyanobacteria, the Fusobacteria and the Tenericutes phyla were less consistently detected within the 6 samples. In three samples (100067, 100069 and 100070), sequences previously reported from environmental metagenome studies were detected. The relative proportion of bacterial sequences according to their phyla taxonomy is displayed for each sample in Figure S1.

### Phage Sequence Assignment

Similarly, phage related sequences were classified according to their best hit sequence in their corresponding family. *Microviridae* and *Siphoviridae* were detected in all skin samples, whereas *Podoviridae* and *Myoviridae* were less systematically identified (Fig. S1).

### Eucaryotic Viruses Sequences Assignment

**Figure 1:**
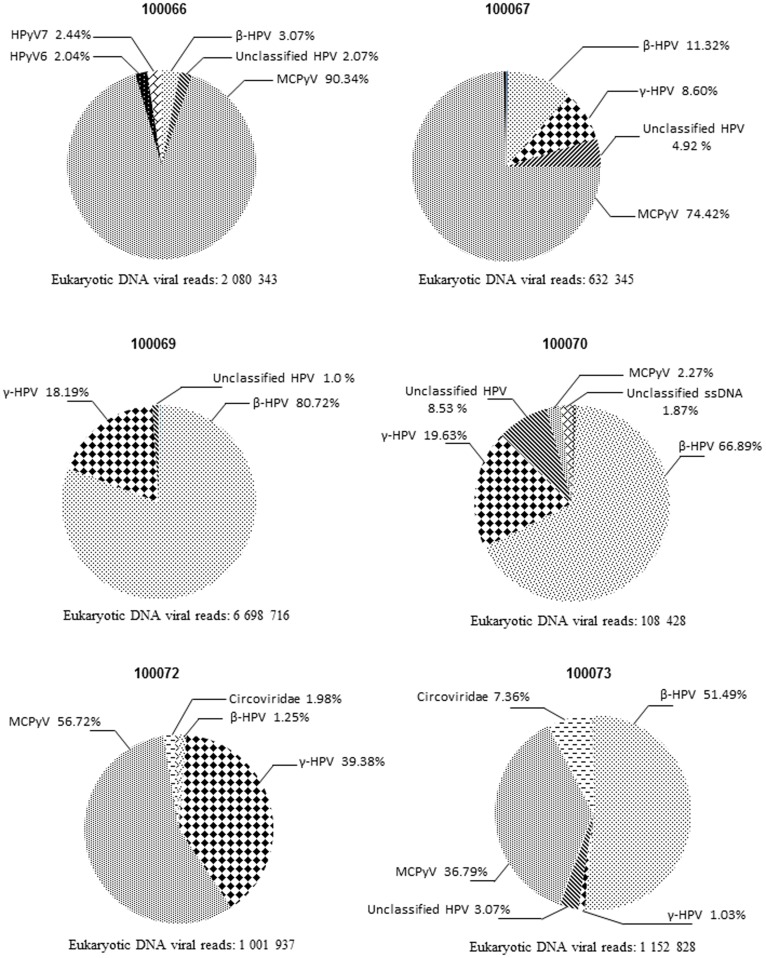
Cutaneous virome. Relative abundance of reads from beta, gamma and unclassified papillomavirus, from circoviruses and other single strand DNA viruses and from the respective polyomaviruses (MCPyV, HPyV6, 7 and 9) within the whole eucaryotic DNA viral reads detected on the skin surface of each sample.

An average of 37.6% (median, 22%; first quartile, 13.9%; third quartile, 73%) of the sequences could be assigned to eucaryotic DNA virus sequences. The detected viral sequences fell into the three predominant *Papillomaviridae, Polyomaviridae* and *Circoviridae* families. In the Papillomaviridae family, sequences were related to the beta- and gammapapillomaviruses whereas Polyomaviridae-related sequences corresponded to Merkel cell polyomavirus (MCPyV), human polyomavirus 6 (HPyV6), human polyomavirus 7 (HPyV7) and human polyomavirus 9 (HPyV9) (Fig. 1).

### Detection and Sequence Analysis of the *Polyomaviridae* Members

**Figure 2:**
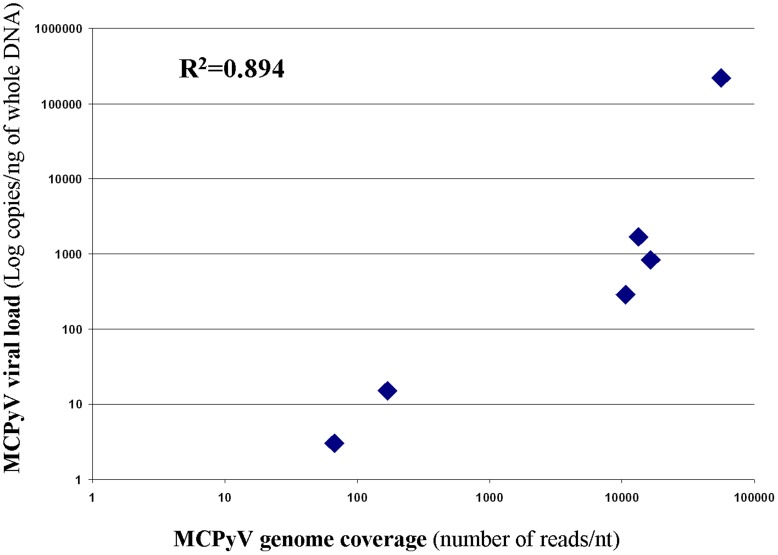
MCPyV genome sequence coverage versus MCPyV viral load in the six samples. Genome coverage is expressed as number of reads per nucleotide and viral load is in genome copies per ng of whole DNA. Coefficient of correlation is: *R^2^* = 0.894; and best fit regression equation is: [MCPyV viral load]  = 4.1 [MCPyV coverage]- 28,850.

Merkel cell polyomavirus sequences were detected and could be assembled as a complete 5kb genome in all 6 samples. Overall coverage (number of reads that encompass each nucleotide) of the MCPyV genome ranged from 60 to 50,000 reads for each base pair. Taking into account the previous results of MCPyV viral load in these samples [11], the HTS coverage was correlated to the viral load (R^2^ = 0.89) (Fig. 2). The 6 strains displayed highly conserved genomic sequences with less than 50 minor single position polymorphisms (data not shown). MCPyV genome sequences were submitted to Genbank with respective accession numbers JQ479315 to JQ479320.

HPyV6 sequences were detected and could be assembled as complete genomes in three samples (100066, 100067 and 100069), whereas HPyV7 complete genome was only assembled from sample 100066 obtained from the patient with MCC. Comparison of the identified HPyV6 and HPyV7 sequences with those available in the databases revealed a very limited polymorphism (<1%). The HPyV7 and 3 HPyV6 genome sequences were submitted to Genbank (accession numbers pending).

Contigs closely related to the African green monkey polyomavirus were detected in the sample 100066. As previously published data [14], the detection of this virus in a skin sample represented an independent confirmation of the newly discovered human polyomavirus 9 (HPyV9) identified by others in the blood of immunnodeficient patients [15].

### Detection and Sequence Analysis of the *Papillomaviridae* Members

The proportion of *Papillomaviridae* related sequences among all viruses ranged from 5% (100066) up to near 100% (100069) of all identified eucaryotic viral sequences (Fig. 1). In order to reach a taxonomic assignment of these *Papillomaviridae* related sequences, an analysis was conducted, focusing on 500 nt contigs encompassing the complete L1 sequence.

Regarding the L1-based taxonomy, an average of 12 (range 6–17) different strains of HPV were identified on skin from the 6 individuals under scope. The highest number of HPV related reads was detected in sample 100066 from the patient with MCC. Regarding sequences homologies, most of the HPV L1 sequences recovered from the 6 samples could be assigned to known HPV species or to new strains of known HPV species. However, 13 L1 complete sequences were probably related to currently unknown HPV species, owing to the fact that these sequences were less than 70% similar to any HPV data base sequences (Table 3).

**Table 3:** Human papillomavirus sequences assembly and assignment in each sample.

Sample	100066					100067					100069			100070			100072			100073		
HPVs family	β-HPV	γ-HPV		uc#		β-HPV		γ-HPV		uc	β-HPV	γ-HPV	uc	β-HPV	γ-HPV	uc	β-HPV	γ-HPV	uc	β-HPV	γ-HPV	uc
N. total Contigs	170	68		81		76		32		12	37	9	15	97	32	20	25	18	26	5	2	2
N. Contigs >500nt	48	3		14		23		6		1	14	4	3	26	18	4	5	6	1	4	2	2
N. Contigs >500nt with partial L1 gene	21	0		6		13		6		1	10	4	0	11	11	3	3	5	0	4	2	2
N. Contigs >500nt with whole L1 cds	17	0		0		9		3		1	6	4	0	7	6	0	3	3	0	4	2	2
**Assignment similarity (based on L1 cds)**																						
<70% ≥70% <90% >90%	0		0		0	0	**3**		0		0	**2**	0	0	**2**	0	0	**2**	0	0	**1**	0
	4		0		0	2	0		1		3	2	0	1	4	0	0	**1**	0	1	1	2
	13		0		0	7	0		0		3	0	0	6	0	0	3	0	0	3	0	0
HPV closest relative (% similarity)	HPV110 (99%)					HPV110 (100%)	**HPV 65 (67%)** *		HPV148 (82%)		HPV80 (83%)	HPV133 (72%)		HPV12 (99%)	**HPV60 (67%)** *		HPV14D (99%)	**HPV65 (66%)**		HPV38b (99%)	**HPV127 (65%)** *	HPV148 (79%)*
	SIBX-3a (98%)					HPV113 (81%)	**HPV112 (62%)** *				HPV110 (100%)	**HPV133 (65%)**		HPV38b (99%)	**HPV121 (65%)**		HPV5 (99%)	**HPV65 (65%)** *		HPV80 (99%)	HPV48 (80%)	HPV148 (71%)*
	SIBX-3a (87%)					HPV150 (99%)	**HPV129 (66%)** *				HPV12 (99%)	**HPV127 (86%)**		HPV75 (99%)	HPV4 (72%)		HPV93 (94%)	**HPV116 (70%)** *		HPV93 (94%)		
	HPV21 (99%)					HPV38b (99%)					HPV150 (81%)	**HPV112 (63%)**		HPV110 (100%)	HPV131 (75%)					HPV124 (78%)		
	HPV36 (99%)					HPV76 (99%)					HPV20 (99%)			HPV80 (86%)	HPV109 (75%)							
	HPV5 (99%)					HPV9 (99%)					HPV120 (85%)			HPV8 (99%)	HPV48 (70%)							
	HPV20 (98%)					HPV96 (85%)								HPV115 (99%)								
	HPV22 (99%)					HPV12 (99%)																
	HPV37 (99%)					SIBX-3a (99%)																
	HPV14D (99%)																					
	HPV122 (76%)																					
	HPV124 (99%)																					
	HPV124 (81%)																					
	FA75 (99%)																					
	HPV24 (97%)																					
	HPV98 (99%)																					
	HPV118 (76%)																					

# uc denotes unclassified HPVs.

* Whole genome sequences confirmed through Sanger sequencing and submitted into Genbank.

New HPV sequences appear in bold and sequences labeled with * are those related to complete genome submitted into Genbank with the JF966371 to JF966379 respective accession numbers (correspondences are displayed in table 4).

**Figure 3:**
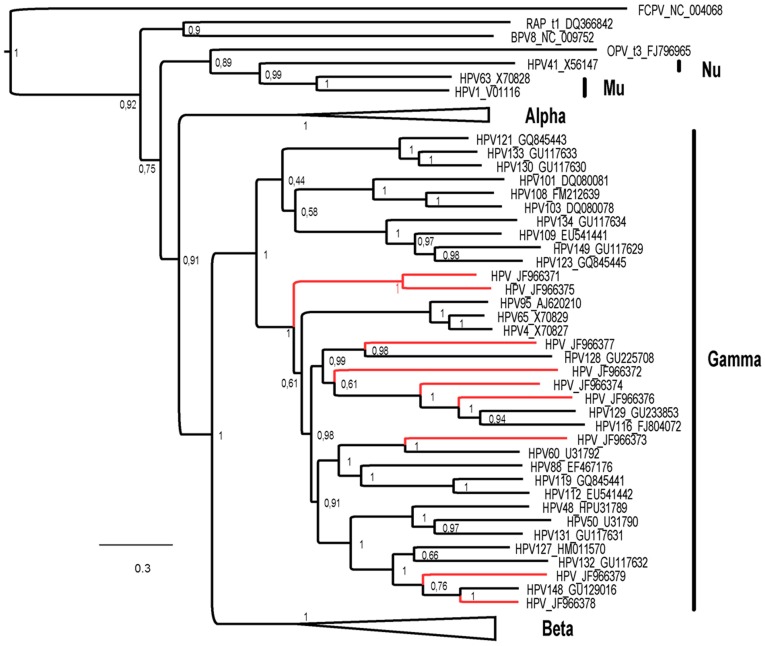
Phylogenic tree of L1 nucleotide sequences. Bayesian phylogeny (using the Tamura-Nei model TN93 with gamma distribution and invariant sites) of the main alpha, beta, gamma, mu and nu papillomaviruses infecting human inferred from their L1 nucleotide sequences (the list of the taxa included all sequences available from the PAVE database and are available at: http://pave.niaid.nih.gov/#prototypes?type=human). The new sequences (red branches) and species reported here belongs to the *Gammapapillomavirus* genus only, therefore, *Alphapapillomavirus* and *Betapapillomavirus* subtrees have been collapsed for clarity reasons. The tree is rooted by a bird *Etapapillomavirus*: the *Fringilla coelebs Papillomavirus* (FCPV). The added animal papillomaviruses to the phylogeny are the Roussetus aegyptiacus papillomavirus type 1 (RAP), the Bovine papillomavirus 8 (BPV8) and the Ovine papillomavirus (OPV) type 3. Posterior probabilities are reported for each node and sequences obtained during this study are depicted by grey/red branches. Each Human papillomavirus is noted HPV followed by the number of the species and by its Genbank accession number.

Among these 13 new HPVs sequences (in bold, Table 3), the distribution of the contigs along the genome allowed the design of primers for additional Sanger sequencing leading to the description of whole genome sequences of 9 new HPV belonging to the gamma group (Accession numbers are respectively JF966371 to JF966379). During the course of our study, new HPV sequences were released and both JF966378 and JF966379 represented new strains of HPV type 148 [8]. Subsequent analysis of the 7 remaining new HPV sequences, have furthermore shown that JF966371 and JF 966375 were two close strains of the same new HPV species, reducing the total number of putative new species reported here to 6. Sequences comparison data of these 9 HPVs with previously known HPV species are displayed in Table 4. The phylogenetic analysis on the L1 protein has confirmed and clarified the global taxonomic assignation obtained from pairwise comparison results and clearly clustered these new sequences within the *Gammapapillomavirus* genus (Fig. 3).

**Table 4:** Genomic organization of 9 new Human gamma-papillomavirus sequences.

GenBank accession number	JF966371*	JF966372	JF966374	JF966373	JF966375*	JF966376	JF966377	JF966378**	JF966379**
Genome lenght (nt)	7300	7299	7251	7167	7286	7219	7265	7152	7095
Most closely related HPV type (accession number)	**HPV 65**(X70829)	**HPV 112**(EU541442)	**HPV 129**(GU233853)	**HPV 60**(U31792)	**HPV 65**(X70829)	**HPV 116**(GU233853)	**HPV 127**(HM011570)	**HPV 148**(GU129016)	**HPV 148**(GU129016)
Nucleotide (amino acid) sequence similarity (%) of L1	66,6 (77.4)	62.5 (73.3)	66.2 (80.3)	67.3 (79.9)	65.5 (77.8)	69.7 (82.1)	65.3 (74.8)	79.2 (92.6)	70.9 (83.0)
E6	103–519 (138)	103–528 (141)	103–627 (174)	103–546 (147)	103–546 (147)	103–528 (141)	103–525 (140)	103–519 (138)	103–519 (138)
E7	521–805 (94)	525–815 (96)	630–926 (98)	521–814 (97)	521–808 (95)	533–823 (96)	522–815 (97)	519–800 (93)	519–80 0 (93)
E1	789–2615 (608)	799–2640 (613)	913–2724 (603)	798–2603 (601)	792–2615 (607)	810–2642 (610)	802–2622 (606)	784–2592 (602)	784–2580 (598)
E2	2557–3750 (397)	2573–3754 (393)	2660–3829 (389)	2539–3750 (403)	2557–3744 (395)	2578–3744 (388)	2558–3766 (402)	2525–3724 (399)	2513–3685 (390)
E4	3023–3508 (161)	–	3033–3590 (185)	3011–3508 (165)	3023–3505 (160)	3041–3508 (155)	3090–3533 (147)	3108–3482 (124)	3096–3443 (115)
L2	3752–5350 (532)	3761–5305 (514)	3831–5333 (500)	3750–5279 (509)	3746–5341 (531)	3747–5273 (508)	3771–5300 (509)	3724–5232 (502)	3687–5180 (497)
L1	5307–6857 (516)$	5316–6872 (518)	5342–6925 (527)	5269–6816 (515)$	5313–6848 (511)	5282–6841 (519)	5248–6858 (536)$	5244–6776 (510)	5191–6717 (508)

For HPV's genes E6 to L1, open reading frame positions and numbering are indicated with the size of the respective predicted protein in brackets.

- $ deduced from the second start codon likely predicted for in vivo use.

- * JF966371 and JF966375 are two strains from the same new HPV species.

- **JF966378 and JF966379 are two strains from the recently described HPV148 species.

### Detection and Sequence Analysis of the *Circoviridae* Members

Sequences belonging to the recently proposed *Cyclovirus* genus were identified in all samples except 100069. We found several sequence reads with similarities to cyclovirus TN9, PK5034, PK5222, but a greater proportion matched to the cyclovirus NG12, which was previously identified in human feces. In two samples (100072 and 100073), we acquired the complete genome of a novel cyclovirus (1779 nt) sharing a nucleotide identity of 79% with cyclovirus NG12. A partial sequence (1154 nt) of this novel genome was also detected in the sample 100070 with a nucleotide identity of 99%. In addition, partial circovirus-like sequences closely related to circovirus-like genomes CB-A and RW-E were identified in the sample 100066.

This identified novel genome displays the typical genomic organization of cycloviruses, with two ORFs in opposite orientation encoding a putative replication associated protein (rep), and a putative capsid protein (cap). This genome also contains a putative stem-loop structure located in the 5' intergenic region and a short 3' intergenic region of eight nucleotides. As for all cycloviruses, the top of the stem-loop displays the highly conserved nonanucleotide motif 'TAATACTAT'. According to the criterion defined by Li et al [16], this sequence represents a new strain of the cyclovirus NG2 proposed species.

Furthermore, specific results regarding other new *Circoviridae* related sequences detected in sample 100072 were published elsewhere with the description of the first human gyrovirus member [17].

## Discussion

Recent advances in molecular technologies have identified a much greater diversity of cutaneous flora than what was revealed using culture-based methods [1], [18]. Bacterial microbiome studies were mostly based on 16S ribosomal RNA metagenomics since the *16S rRNA* gene contains both consensus regions targeted by PCR and variable regions allowing direct taxonomic assignment whenever possible and phylogenetic studies if required. In the present study, in order to characterize the skin viral microbiota, HTS, a highly comprehensive method based on random sequencing of the entire DNA present in a given sample was applied to superficial skin samples collected by cutaneous swabbing. This method was previously successfully used for studying environmental media, intestinal microbiota and, more recently, human blood samples [19]–[22]. However, HTS requires large amounts of DNA and comprehensive reference genome sequences for microbial populations, a prerequisite that might raise some concern regarding its application to skin samples. In our study, difficulties regarding the template DNA amount in skin swab samples were circumvented by a first round of whole genome amplification using multiple displacement amplification (MDA), a technique known for its ability to introduce low bias for linear DNA amplification [23]. Nervertheless, this method may favor the amplification of circular molecules which might have led to an overestimated proportion of circular DNA genomes (*Polyomaviridae, Papillomaviridae, Circoviridae*) as illustrated by the high proportion of eucaryotic viral sequences detected in the two samples 100066 and 100069. Eventually, sequences analysis was restrained to taxonomic assignment with no attempt to obtain any data regarding functional analysis. Also, the contribution of each species was not corrected by genome sizes: such standardization would have lowered by several orders of magnitude the contribution of bacterial species compared to viral species. Thus, comparison of frequencies should be made within bacteria or viruses.

Taxonomic assignment of bacterial sequences detected in our study confirms previous data derived from 16S rRNA analysis. Indeed, most of the bacterial sequences belonged to four dominant phyla, Actinobacteria, Firmicutes, Bacteroidetes and Proteobacteria. Previous molecular analysis of the skin bacterial microbiota have emphasized that bacterial diversity depends on the body site with additional interpersonal and temporal variability [24]. More specifically, surface areas could be divided in dry, moist or sebaceous environments regarding skin physiology and it was demonstrated that these conditions are likely to influence the composition of the bacterial microbiome [25]. In our study, inter-individual variations are obvious when considering the relative proportion of bacterial sequences within the 6 skin samples tested (Fig S1). The samples were all collected from the face of the patients, which can be considered as a sebaceous environment, and *Propionibacterium* and *Corynebacterium* spp (*Actinobacteria*)-related sequences, theoretically expected in such an environment, were indeed detected. Additionally, our study identified numerous sequences from *Staphylococcus* spp and *Streptococcus* spp (Firmicutes) and *Proteobacteria* phylum that are more likely to be found in dry and moist skin environments respectively [18], [24]. This discrepancy may be explained by technical limitations of 16S rRNA studies with variable performances according to the phyla under scope whereas HTS metagenomic randomly targets all genomic material with a higher sensitivity and is probably less liable to such variations. In addition, the domination of the 16S based criteria in bacterial taxonomy compared to other molecular characters to date less represented in databases may also contribute to this observed discrepancy.

Detection of microorganisms on human skin is not restricted to bacteria since viruses, fungi and some acarids like *Demodex* spp are present on skin of healthy individuals. Most described fungal organisms identified on healthy skin are related to *Malassezia* spp whereas *Candida* spp is infrequently detected on normal human skin, contrasting with mucosal membrane where this yeast is physiologically present in limited amounts [26]. These concepts are supported by our study in which many reads corresponded to *Malasseziales* species whereas *Saccharomycetales* related reads were rare.

The presence of archaea on the skin has not been previously reported but they might have been missed by 16S rRNA sequencing. Conversely, our results suggest that archaea sequences can be detected on skin surface although the low number of reads raise concerns about the relevancy of these data that need to be confirmed by further investigations. Alternatively, since superficial skin is permanently exposed to surrounding environments as illustrated by the detection of numerous reads related to sequences previously reported from environmental metagenomic studies, we could not rule out contamination by environmental sequences or DNA present in the reagents. Interestingly, sequences from *Escherichia coli* and *Pseudomonas spp* species that are in our experience common contaminants of our pipeline did not contribute significantly to the results, most likely because of the abundance of the skin microbiome.

Among viral sequences, only few reads were related to phages. The relative proportion of the various phage species in the 6 samples is in keeping with the spectrum of their bacterial hosts usually present on healthy skin surface. This is in accordance with the detection of bacteriophages in a recently reported virome study conducted in HIV patients peripheral blood, a result likely to be associated to the presence of circulating bacteria in this setting [21]. However, the diversity of the phage sequences identified in our study is of great interest because these viruses are involved in lateral gene transfer that can influence the bacterial diversity but also some of the properties of bacteria including virulence and resistance to antibiotics.

According to our data, eucaryotic DNA viruses detected on skin samples displayed a high diversity including various representatives of *Papillomaviridae*, *Polyomaviridae* and *Circoviridae*. However, as mentioned above, some technical limitations must be pointed out that might have reduced a full appraisal of this diversity. First, the initial DNA enrichment step through phi29 MDA might have favored amplification of circular DNA genome as suggested by the recovery of some plasmid sequence. Furthermore, the procedure of DNA extraction and amplification of skin swabs did not allow the recovery of RNA virus genomes. Eventually, as swab samples were all collected from the same facial area, skin site variations, which are known to influence bacterial microbiota, could not be investigated.

Viral colonization of healthy skin was primarily and extensively described for papillomaviruses and the commensal nature of these viruses is now a widely admitted data. Indeed, the presence of resident HPVs on normal skin was reported for both Beta- and Gammapapillomaviruses [27], [28]. Our results confirm these data and provide evidence for an asymptomatic carriage of numerous HPV strains (up to 17) on each individual skin sample. This skin carriage may reflect a viral shedding from cutaneous micro-reservoirs that might be hair bulbs, since such high prevalence and multiplicity was mainly described in forehead hair bulb studies [29]. Furthermore, we identified 13 new Gamma-HPV strains including 9 strains for which the whole genome sequence was further confirmed by the Sanger method allowing the description of 6 new HPV species (Fig. 3). This suggests that the diversity of the resident cutaneous gamma-HPV group might be actually larger than previously described [30]. Chronic asymptomatic shedding of HPVs from skin surface is a well-known feature and most of these viruses are considered as innocent bystanders with no significant tissular damages resulting from their replication which is mainly supported by keratinocytes differentiation.

A similar chronic carriage and shedding at the surface of healthy skin appears to be a hallmark of human polyomaviruses with cutaneous tropism as well. Such a viral ecosystem was suggested by previous reports on MCPyV, HPyV6 and HPyV7 [12], [13] but no systematic metagenomic study was carried out with high performance methods on normal-appearing skin. In the present study, a correlation was observed between the amount of reads and the MCPyV viral load (evaluated by qPCR in original samples), as illustrated by the relative coverage rates of assembled whole genomes from the six skin samples under scope (Fig. 2). This observation supports the concept of metagenomic approaches being broader and at least as sensitive as specific target amplification and allowing an exhaustive coverage if the corresponding sequences are present at sufficient levels [31]. Of note, these results were obtained with a mean depth per sample of 8.6×10^6^ reads, which is far lower than the depth currently permitted by the improvements of Illumina technology that can reach 10^8^ reads. Whole genome comparison of the six MCPyV strains described in our study revealed high sequence similarities to each others and the strain derived from the healthy skin of the MCC patient did not present any specific characteristic. Furthermore no genomic mutation nor deletion were identified suggesting the likely episomal nature of the viral sequences detected in all cases.

Detection of both HPyV6 and HPyV7 sequences in respectively 3 and 1 sample(s) is in keeping with previous studies that have already detected these viruses on superficial layers of healthy skin [12]. Accordingly, both of them appear to be less frequently detected than MCPyV and, unlike MCPyV, no skin disease has been associated with these two new viruses to date [12], [32], [33]. As for MCPyV, full genome sequences of HPyV6 and HPyV7 identified in this study only demonstrated minor single nucleotide polymorphisms when compared to published data. This low diversity of HPyVs species that appears more limited in its extent than that of HPVs, needs to be confirmed by the analysis of larger cutaneous sample set.

The presence of numerous *Circoviridae* representatives on the superficial layers of the skin can be expected since these viruses are highly ubiquitous. These data are strikingly reminiscent of the situation of the very close *Anelloviridae* family including TT virus and TTV-like mini virus that are frequently detected in various body sites [34]. In our study, the detected sequences are related to the *Cyclovirus* genus. Cycloviruses infect mainly animals with likely cross species transmissions [35]. Animal cycloviruses could be also detected in feces of primates including humans [16] which suggests that the specificity of the detection of cycloviruses on the skin remains to be established.

The biological role of such a highly polymorphic viral microbiota on skin surface is largely hypothetical and has been much less extensively studied than for its bacterial counterpart. Skin bacterial populations are either transient or symbiotic and are likely to provide protection against more virulent organisms, thus resulting in a function of surrogate immune sentinel. A similar function of skin viral microbiota may be hypothesized but warrants to be further investigated. Indeed, resident viruses may act protectively against other bacterial, fungal or viral pathogens through either direct (phages) or indirect (by maintaining a high level of efficiency of skin immune cells) pathways. Similarly, the influence of disturbances in skin bacterial and fungal populations has been emphasized in skin disorders involving local skin immune system dysregulation including seborrheic dermatitis, acne, atopic dermatitis, or psoriasis [36], [37], and the influence of such variations of the viral flora on skin homeostasis and alterations may also be worth considering. Although most cutaneous HPVs and the more recently identified cutaneous HPyVs can be considered as resident symbiotic organisms on normal skin, some of them are associated with skin diseases like HPV5 or HPV8 or HPV50 involved in epidermodysplasia verruciformis or squamous cell carcinoma [38]. Also, numerous HPVs and MCPyV are associated to pre-malignant and malignant skin conditions in immunocompromised as well as in immunocompetent patients [39], [40]. The hypothesis of the viral skin flora being affected by the immune status of the host is consistent with our experimental data featuring a higher viral diversity of both HPV and HPyV species in the patient with MCC, a rare malignancy mostly affecting elderly and immunocompromised populations [41]. Indeed, despite the limited number of cases under scope precluding any statistical analysis, 17 different HPV strains as well as most of the HPyVs previously suspected to have a cutaneous tropism (MCPyV, HPyV6, HPyV7 and HPyV9 ) were identified only on the MCC patient's skin, suggesting that the relationship between skin conditions and/or immunologic status on one hand and the skin virome composition on the other hand should be further investigated.

In summary, this pioneer study is the very first one that applies highly powerful HTS to the description of the viral skin microbiota. Despite a limited number of patients under scope, it demonstrated an unexpected high diversity of DNA viruses on the normal appearing skin with a large interpersonal variability. These viruses are represented essentially by various species of betapapillomaviruses and gammapapillomaviruses, polyomaviruses and circoviruses. Although viruses are generally considered as pathogen agents, our findings highlight the complex viral flora at the surface of the non pathological skin of all individuals. The dynamics and anatomical variations of the skin viroma and their potential variations according to pathological conditions remain to be further studied. It should be also investigated if these viruses, alone or in combination, could represent potential triggers of skin cell proliferation and oncogenesis.

## Materials and Methods

### Cutaneous Samples and DNA Extraction

Cutaneous samples were obtained by swabbing normal-appearing skin of the face from white individuals. A wood cotton tip swab was applied thoroughly along the forehead without any prior specific skin care. Skin samples were obtained in 5 healthy people with no history of any skin conditions (Patient 100067, male, 32 years old; patient 100069, female, 53 years old; patient 100070, female, 58 years old; patient 100072, female, 63 years old and patient100073, female, 55 years old) and in one patient with a previous MCC lesion localized on the buttock (Patient 100066, male, 75 years old). These skin specimens were initially obtained in spring 2009 to compare MCPyV strains detected on the normal skin from healthy subjects and a patient with MCC [11]. Swabs were suspended in 400 µL of phosphate buffer. DNA was extracted from 300 µL of this suspension with an automatic EasyMag apparatus (BioMérieux, Marcy l’Etoile, France) and amplified by the bacteriophage phi29 polymerase based multiple displacement amplification (MDA) assay using random primers. The reaction was performed with the REPLI-g Midi kit (Qiagen), according to the manufacturer's instructions.

### High Throughput Sequencing

Sequencing was conducted on an Illumina® HiSeq-2000 sequencer (GATC Biotech AG, Konstanz, Germany). 5 µg of high molecular weight DNA resulting from isothermal amplification was fragmented into 200 to 350 nt fragments, to which nucleotide adapters including a tag, allowing for multiplexing several samples per lane or channel, were ligated.

### Sequence Analysis

Sequences were first selected or trimmed according to their quality scores. As previously described [30], the human genome was filtered with SOAPaligner (http://soap.genomics.org.cn) using the *Homo sapiens* hg19 reference. A number of assembly programs dedicated to short or medium-sized reads were used to generate contigs : Velvet (http://www.ebi.ac.uk/), SOAPdenovo (http://soap.genomics.org.cn/) and CLC Genomics Workbench (http://www.clcbio.com). The comparison of the single reads and contigs to already known genomic and taxonomic data was done on the generalist nucleotidic (nt) and proteic (nr) databases maintained locally. The aforementioned databases were scanned using the BlastN and BlastX algorithms provided by Paracel Blast (Striking Development), a software capable of executing searches on multiple non-shared-memory processors simultaneously. Binning (or taxonomic assignment) was based on the best hit among reads with a significant e-value (generally 10^−3^).

### Phylogeny

The phylogenetic analysis of the Human papillomaviruses L1 sequences was performed with 145 published sequences obtained from the PAVE database and listed at: http://pave.niaid.nih.gov/#prototypes?type=human. Sequences were aligned using Mega5 [42] and the Muscle algorithm [43]. After the cut off of ambiguously aligned beginning and end of the matrix, there were a total of 1374 positions in the final dataset. The evolution model describing the best the substitution pattern was selected using Mega5 package [42]. Non-uniformity of evolutionary rates among sites were considered by using a discrete Gamma distribution (+G) with 5 rate categories and by assuming that a certain fraction of sites are evolutionarily invariable (+I). The phylogeny resulted from a bayesian analysis implemented in the BEAST software [44].

The tree was rooted by a bird papillomavirus that represent an adequate root as birds papillomaviruses clearly cluster from mammals papillomaviruses and because birds and mammals have distinct phylogenetic origins. A few others animal papillomaviruses have been added to the phylogeny to verify the robustness of the ingroup and confirm, in the context of our analysis, the monophily of alpha, beta and gammapapillomaviruses.

## Supporting Information

Figure S1 **Bacterial and phage microbiome.** Tables A and B represent respectively bacterial and phage assignments with relative abundance based on reads number.
(DOCX)
