# Supplementary material for: Human Skin Microbiota: High Diversity of DNA Viruses Identified on the Human Skin by High Throughput Sequencing
Source: PLoS One. 2012 Jun 19;7(6):e38499. doi: 10.1371/journal.pone.0038499 (PMC3378559; doi:10.1371/journal.pone.0038499)
Supplement: Figure S1 — Bacterial and phage microbiome. Tables A and B represent respectively bacterial and phage assignments with relative abundance based on reads number. (DOCX) [file pone.0038499.s001.docx]

(A)

| Sample |  | **100066** | **100067** | **100069** | **100070** | **100072** | **100073** |
| --- | --- | --- | --- | --- | --- | --- | --- |
| **Bacterial reads** |  | 658 075 | 2 766 794 | 672 919 | 900 033 | 2 876 654 | 3 537 704 |
| **Phylum** |  |  |  |  |  |  |  |
| Actinobacteria |  |  |  |  |  |  |  |
|  | Corynebacterium sp. | 0.12 % | 6.51 % | 5.09 % | 0.65 % | 0.35 % | 0.95 % |
|  | Propionibacterium sp . | 0.03 % | 0.16 % | 19.27 % | 0.49 % | <0.01 % | 0.06 % |
|  | Other Actinobacteria | 0.04 % | 2.94 % | 1.17 % | 4.13 % | 0.13 % | 0.80 % |
| Bacteroidetes |  | 0.99 % | 8.93 % | 3.04 % | 13.17 % | 0.94 % | 2.36 % |
| Cyanobacteria |  | 0.06 % | 3.08 % | 1.14 % | 2.84 % | 0.20 % | 0.14 % |
| Firmicutes |  |  |  |  |  |  |  |
|  | Staphylococcus sp. | 2.81 % | 19.45 % | 22.77 % | 17.90 % | 18.42 % | 48.31 % |
|  | Streptococcus sp. | 26.49 % | 1.95 % | 7.29 % | 3.12 % | 2.39 % | 7.76 % |
|  | Other Firmicutes | 56.52 % | 12.90 % | 9.09 % | 8.83 % | 35.74 % | 13.51 % |
| Fusobacteria |  | 0.15 % | 0.11 % | 0.26 % | 0.31 % | 0.08 % | 0.02 % |
| Proteobacteria |  | 11.88 % | 40.42 % | 30.14 % | 41.65 % | 41.51 % | 25.73 % |
| Tenericutes |  | 0.79 % | 1.27 % | 0.23 % | 0.12 % | 0.04 % | 0.23 % |
| Environmental samples |  | 0.07 % | 2.16 % | 0.40 % | 6.51 % | 0.08 % | <0.01 % |
| Other bacteria |  | 0.05 % | 0.15 % | 0.12 % | 0.32 % | 0.11 % | 0.12 % |

Nd : Not detected

(B)

| Sample | **100066** | **100067** | **100069** | **100070** | **100072** | **100073** |
| --- | --- | --- | --- | --- | --- | --- |
| **Phage reads** | 235 | 12 167 | 187 | 840750 | 14 746 | 41 532 |
|  |  |  |  |  |  |  |
| Myoviridae | 5.53 % | 2.80 % | 19.25 % | 0.01 % | Nd | 0.24 % |
| Siphoviridae | 25.11 % | 7.21 % | 48.66 % | 1.43 % | 75.92 % | 1.58 % |
| Microviridae | 34.47 % | 66.59 % | 25.13 % | 89.63 % | 23.39 % | 98.13 % |
| Podoviridae | 0.43 % | 0.21 % | 1.07 % | <0.01 % | 0.44 % | Nd |
| Environmental samples | 22.98 % | 15.46 % | 1.07 % | Nd | 0.16 % | Nd |
| Inoviridae | Nd | 0.16 % | Nd | Nd | Nd | 0.02 % |
| Unclassified phages | 11.49 % | 7.57 % | 4.81 % | 8.92 % | 0.09 % | 0.03 % |

Nd : Not detected
